# Plasmodium Vivax Can Only Be Responsible for GBS if All Other Triggers Have Been Ruled Out

**DOI:** 10.1002/ccr3.72546

**Published:** 2026-04-19

**Authors:** Josef Finsterer

**Affiliations:** ^1^ Neurology Department Neurology & Neurophysiology Center Vienna Austria

**Keywords:** Guillain‐Barre syndrome, immunoglobulins, malaria, nerve conduction studies, plasmodium vivax

To the Editor,

We read with interest the article by Abdela et al. about a 21‐year‐old man with Guillain–Barré syndrome (GBS) that developed 5 days after infection with Plasmodium vivax [1]. Due to the temporal relationship between the infection and GBS, a causal link between the two was assumed [1]. The patient recovered almost completely by Day 54 with intravenous immunoglobulin treatment [1]. The study is interesting, but some points require discussion.

The first point is that alternative triggers for GBS have not been sufficiently ruled out. The most common triggers of GBS include *Campylobacter jejuni* , followed by infections with Hemophilus influenza, *Mycoplasma pneumoniae* , and the Zika virus [2]. Other, less common triggers of GBS include SARS‐CoV‐2, HIV, RSV, HBV, HCV, HAV, HEV, EBV, CMV, HSV, measles virus, enterovirus, influenza A virus (H1N1), chikungunya virus, parecho virus, Toscana virus, Japanese encephalitis virus, varicella zoster virus, Plasmodium falciparum, Leptospira, *Orientia tsutsugamushi* , syphilis, and dengue. Noninfectious triggers of GBS include vaccinations (e.g., against SARS‐CoV‐2, influenza, polio, rabies, hepatitis A, B, C, D, E), lymphomas, kidney transplants, intravenous administration of gangliosides, and trauma [2]. Before Plasmodium vivax can be considered as the cause of GBS in the index patient, these alternative triggers must be thoroughly ruled out. The exclusion of differential causes of GBS should not be based on the medical history, as in the index case, but on the results of objective serological, imaging, and functional tests.

The second point is that no nerve conduction studies were performed. Therefore, it was not possible to determine which subtype of GBS the patient had [1]. These include acute inflammatory demyelinating polyneuropathy (AIDP), acute motor axonal neuropathy (AMAN), Miller Fisher syndrome (MFS), the cervico‐pharyngeal‐brachial (CPB) subtype, cranial nerve neuropathy, and Bickerstaff encephalitis (BBE). Knowledge of the GBS subtype is crucial, as treatment and progression can vary significantly depending on the subtype. With regard to the subtypes, we disagree with the view expressed in the introduction that GBS is only a demyelinating polyneuropathy [1]. GBS can also be of the axonal type or affect only the autonomic fibers, which have different triggers and different outcomes.

The third point is that the patient obviously had bulbar involvement (cranial nerves 9 and 10), but no impairment of other cranial nerves was reported [1]. Were other cranial nerves affected besides the 9th and 10th cranial nerves? Since Table 1 mentions that there was weakness of the orbicularis oris muscle on Day 54 [1], there must be involvement of the 7th cranial nerve. Was the involvement of the facial nerve already detected at the onset of GBS, or did it develop later in the course of the disease?

The fourth point concerns the discrepancy between the statement in the case description that the neurological examination at the follow‐up examination after 4 weeks was normal and the information in Table 1 that on Day 26 the patient could only stand with support and suffered from dysarthria [1]. This discrepancy should be clarified.

The fifth point refers to the discrepancy between the statement that the patient suffered from dysarthria and the information in Table 1 that audible sounds were detected on Day 15, suggesting that the patient previously suffered from aphonia. Since aphonia and dysarthria are different clinical pictures, this discrepancy should be clarified.

The sixth point is that the incubation period of Plasmodium vivax is 12 to 18 days or longer [3], which is why a causal relationship between malaria and GBS remains questionable. If the index patient was already infected weeks before the onset of GBS, a causal relationship is rather unlikely.

Finally, we should know why the patient was admitted to the intensive care unit but did not require artificial ventilation. Was there respiratory failure that could only be treated with oxygen or a CPAP mask? It should also be clarified why dysarthria was a contraindication for performing spirometry.

In summary, all alternative triggers for GBS must be ruled out before GBS can be attributed to malaria. It is also crucial to determine the GBS subtype, as treatment, course, and outcome may vary depending on the subtype.

## Author Contributions

**Josef Finsterer:** conceptualization, data curation, formal analysis, resources, validation, writing – original draft, writing – review and editing.

## Funding

The author has nothing to report.

## Ethics Statement

The author has nothing to report.

## Consent

The author has nothing to report.

## Conflicts of Interest

The author declares that the research was conducted in the absence of any commercial or financial relationships that could be construed as a potential conflicts of interest.

## Data Availability

All data are available from the corresponding author.
